# Clinical and psychological phenotypes of type 1 diabetes and disordered eating derived from a case vignette series: T1DE phenotypes

**DOI:** 10.1007/s00125-026-06756-9

**Published:** 2026-05-26

**Authors:** Divina Pillay, Nadine Paul, Amy Harrison, Natalie Zaremba, Jennie Brown, Emmanouela Konstantara, Pil Lindgreen, Jacqueline Allan, Diane Turner, Miranda Rosenthal, Janet Treasure, Khalida Ismail, Marietta Stadler

**Affiliations:** 1 Department of Psychological Medicine, Diabetes, Psychology and Psychiatry Research Group, King’s College London, London, UK; 2 Department of Diabetes, School of Cardiovascular and Metabolic Medicine and Sciences, King’s College London, London, UK; 3 Department of Psychology and Human Development, University College London, Institute of Psychiatry, London, UK; 4 Florence Nightingale Faculty of Nursing, Midwifery & Palliative Care, King’s College London, London, UK; 5 Department of Medical Psychology, Radboud University Medical Center, Nijmegen, the Netherlands; 6 Diabetes Centre, King’s College Hospital, London, UK; 7 Steno Diabetes Center Copenhagen, Department of Prevention, Health Promotion, and Community Care, Copenhagen, Denmark; 8 Institute of Psychiatry, Psychology and Neuroscience, King’s College London, London, UK

**Keywords:** Disordered eating, Eating disorder, Mental health, Type 1 diabetes

## Abstract

**Aims/hypothesis:**

Type 1 diabetes and disordered eating (T1DE) is a common, complex comorbidity of type 1 diabetes with high morbidity and mortality risk. T1DE currently lacks a clear definition, diagnostic differentiation into phenotypes and clinical severity grading, which results in exclusion of people with T1DE from accessing appropriate treatments to improve outcomes.

**Methods:**

We developed anonymised T1DE case vignettes from the baseline assessments of the Safe management of people with Type 1 diabetes and EAting Disorders studY (STEADY) and the London-T1DE pilot service at King’s College Hospital for people with diabetes and severe eating disorder, integrating detailed biomedical and psychiatric data. In an iterative process, phenotypes of T1DE were developed, the T1DE vignettes were sorted into the different phenotypes and a clinical severity grading framework was developed.

**Results:**

The 70 participants (94.3% women) were 34.5 ± 10.6 years old with a diabetes duration of 18.4 ± 12.5 years and HbA_1c_ of 84.8 ± 32.9 mmol/mol (10.2 ± 3.0%). All but one fulfilled ICD-10/Diagnostic and Statistical Manual of Mental Disorders, 5th Edition criteria for a formal eating disorder diagnosis, 34 met criteria for other specified feeding or eating disorder, 27 for bulimia nervosa, six for anorexia nervosa and two for binge eating disorder. Over 50% had a mood disorder, 44.3% an anxiety disorder and 20.0% a trauma-related disorder. From the anonymised T1DE vignettes generated, three main T1DE phenotypes with subtypes emerged: insulin-omission (diabulimia) T1DE (28 participants, 40.0%), characterised by near total insulin omission in intention to lose weight, with (binge–)purge, anorectic and cyclical subtypes; restrict T1DE (17, 24.3%), characterised by restriction of food/carbohydrates (with [binge–]purge, anorectic and hypo subtypes); and binge T1DE (25, 35.7%), characterised by subjective or objective binge eating with or without omission of bolus insulin (with binge–purge, binge-only and hypo subtypes). Severity grading categorised 19 cases (27.1%) as ‘low’, 23 (32.9%) as ‘moderate’ and 28 (40.0%) as ‘severe’ clinical risk.

**Conclusions/interpretation:**

The three T1DE phenotypes will serve as a basis for more precise clinical diagnosis and severity grading and will be used to develop future diagnostic screening tools, improved psychological interventions and integrated treatment care pathways to match the individual clinical presentation and severity in a personalised medicine approach.

**Graphical Abstract:**

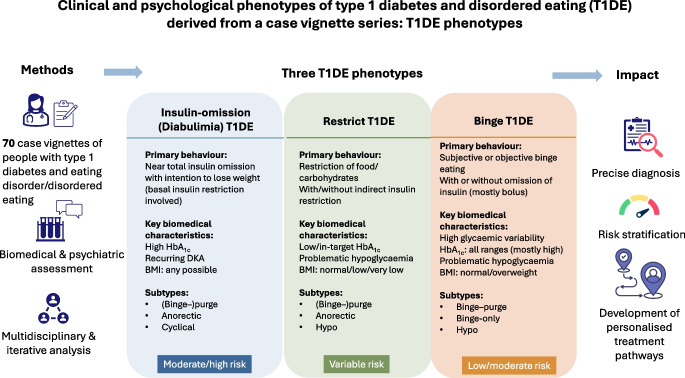

**Supplementary Information:**

The online version contains peer-reviewed but unedited supplementary material available at 10.1007/s00125-026-06756-9.


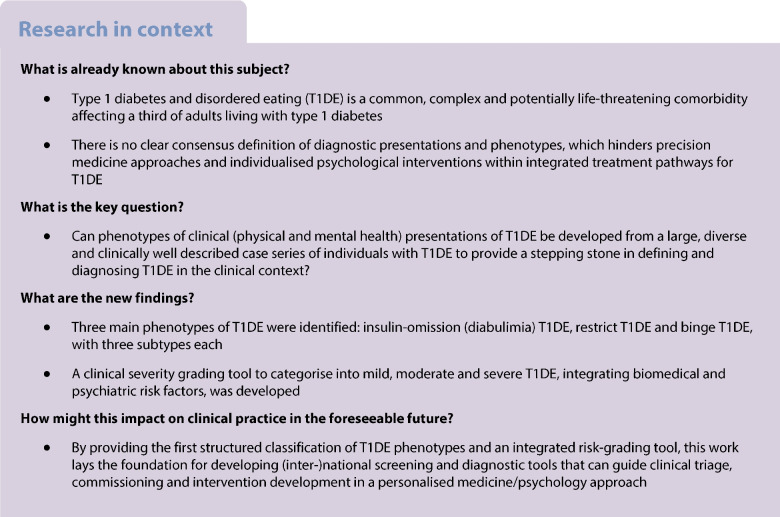


## Introduction

Eating disorders are twice as common in people with type 1 diabetes mellitus when compared with the general population [1]. Disordered eating, including diabetes self-care behaviour impacted by disordered eating cognitions, affects up to two-thirds of the adult clinic population living with type 1 diabetes [2, 3]. The complex relationship between type 1 diabetes and eating disorders/disordered eating has been described in theoretical models, illustrating the intertwining of both conditions: pre-existing eating disorder is aggravated by type 1 diabetes; disordered eating may exacerbate into clinically relevant eating disorders, triggered by the onset of type 1 diabetes which is usually preceded by significant unintentional weight loss due to the catabolism caused by insulin deficiency and rapid weight regain when commencing treatment; type 1 diabetes self-management puts a relentless focus on food intake (carbohydrate counting) and numbers (glucose monitoring); iatrogenic triggers, such as regular weighing in clinics and judgmental consultations on ‘diabetes control’; adverse experiences with the condition type 1 diabetes (hospital admissions, severe hypoglycaemia, long-term complications) in combination trigger, fuel and further entrench the disordered eating/eating disorder behaviours [4–6]. The median adult age of onset of type 1 diabetes in the mid-twenties may coincide with a vulnerable phase of life transitions (e.g. adolescence, leaving home or pregnancy) [7, 8]. Metabolic optimisation using hybrid closed loop (HCL) insulin pump systems can drive initial weight gain [9] and changes in eating behaviours [10]. Anecdotally, people with type 1 diabetes have found that constant access to their glucose readings on continuous glucose monitoring systems has contributed to restrictive eating patterns [11, 12].

As there is no ICD-10 (5) or Diagnostic and Statistical Manual of Mental Disorders, 5th Edition (DSM-5) coded diagnosis to encompass the combination of type 1 diabetes and disordered eating/eating disorders, individuals have been previously labelled as having ‘diabulimia’ or ‘brittle diabetes’, or being ‘non-compliant’ or ‘non-adherent’, which excludes them from getting help, which further increases feelings of isolation, hopelessness and secrecy [6, 13]. The term ‘type 1 diabetes and disordered eating (T1DE)’ has been proposed in the context of a national guideline by the Royal College of Psychiatry, UK, in a research trial (STEADY) and clinical service development of the King’s College London group as a working definition for diagnostic criteria [14]. The umbrella term T1DE includes: (1) eating disorder (ICD-10 or DSM-5 diagnosable) plus type 1 diabetes; and (2) disordered eating behaviour and cognitions impacting on diabetes self-care (e.g. insulin omission, insulin restriction, food restriction), often driven by a pervasive fear of insulin as a cause of weight gain in the context of type 1 diabetes and covering a broad spectrum of clinical presentations [14, 15]. High physical health risk in conventionally defined eating disorders in the general population is recognised, with anorexia nervosa having the highest mortality rate [16, 17]. The comorbidity with type 1 diabetes increases risk further, with added risks of acute and long-term diabetes complications. Mortality risk and morbidity is high in this group of people, with a threefold higher mortality rate reported in women with type 1 diabetes with comorbid eating disorders/disordered eating restricting insulin compared with people with type 1 diabetes not restricting insulin [18]. Due to lack of definition of the condition T1DE, larger population-based observational studies on outcomes and risk predictors are missing.

The challenge in defining and developing treatment pathways for disordered eating/eating disorders in the context of living with type 1 diabetes lies in capturing the complex psychology and physiology of the different features of subtypes of T1DE. While fear of gaining weight is a driver found in eating disorders within both the general population and people with type 1 diabetes, T1DE can in some presentations be more centred on insulin omission/restriction than on food restriction. Therefore, the usual screening and diagnostic tools used in eating disorders may under- or over-detect disordered eating in this population as type 1 diabetes-related factors are not considered. Body image and weight concerns may prevent people with T1DE from engaging with their diabetes self-care and lead to diverse and varying behaviours (e.g. not taking insulin to cover food, not taking insulin at all to induce weight loss, avoiding eating to treat hypoglycaemia, binge eating, restriction of food intake/calories or carbohydrates to affect weight and insulin requirements) [5, 6], resulting in an increased risk of complications [18, 19].

The T1DE pilot service at King’s College Hospital for adults with diabetes and severe eating disorder integrates medical management of type 1 diabetes with psychiatric in- and outpatient care and improved glucose levels and diabetes acute complications rates in the group accessing the T1DE-service and the psychotherapies offered [14]. The Safe management of people with Type 1 diabetes and EAting Disorders studY (STEADY) is a novel complex intervention developed using experience-based co-design within the theoretical framework of cognitive behavioural therapy (CBT) and diabetes education [20]. STEADY is designed for adults with clinically mild to moderate presentations of T1DE (who were well enough to take part in an RCT with a ‘usual care’ control arm) [20] and was tested in a feasibility RCT [15]. While conducting clinical diagnostic assessments of people with T1DE in the STEADY trial [15] and London-T1DE [14], it was noted that the current diagnostic classification framework of DSM-5 [21] and ICD-11 lacked a specific and nuanced method to capture the clinical presentations of T1DE. While some people could be captured by the existing eating disorder diagnoses, they would often fall into categories of bulimia nervosa or other specified feeding or eating disorder (OSFED), which felt inadequate and inappropriate to reflect the complexity of the symptom profiles, the clinical risk and the nuances of the interplay between diabetes and disordered eating.

Both studies [14, 15] included detailed physical and mental health assessment at baseline and provided the data for generating a series of T1DE case vignettes to describe the multifactorial aetiology and diverse clinical presentations of T1DE. We aimed to analyse and synthesise these case vignettes to develop definitions for T1DE clinical phenotypes and severity stages for adults living with type 1 diabetes and disordered eating/eating disorder, rooted in real-world clinical evidence. The aim is to provide a basis for refined diagnostic and care pathways for personalised medicine approaches for different phenotypes of clinical presentations of T1DE.

## Methods

This mixed-methods study is embedded in two clinical research projects developing complex interventions for T1DE: STEADY [15] and T1DE-London [14]. We collated the data from a combined series of 70 cases of T1DE, ranging from the mild to moderate spectrum (STEADY, *n*=43) to the moderate and very severe spectrum (T1DE-London, *n*=27).

### Participants

#### STEADY

Participants of the STEADY feasibility RCT who attended a baseline visit with structured medical and psychiatric assessment (*n*=43) were included in this analysis (ClinicalTrials.gov registration no. NCT05140564).

#### T1DE-London

Service users of T1DE-London were approached for consent to have their clinical records, including psychiatric and psychotherapeutic baseline assessments [14], analysed along a similar data collection framework as was used for STEADY baseline data collection (REC-reference: 21/PR/1400); 27 out of 76 service users consented to take part.

### Case vignette generation and analysis

Case vignettes are abbreviated and fully anonymised short case summaries that include the key medical, psychiatric, psychological and anthropometric data (ethnicity and sex/gender were self-reported), while maintaining anonymity. Additional changes were made, so the participants would not immediately recognise themselves in the case vignette series, by omitting specifics (e.g. profession, age adjusted within the decade, not specifying type of illicit drug use). The vignettes have been proofread by collaborators with lived experience. Data collection for the case vignette generation was as follows: STEADY: data from the Structured Clinical Interview for DSM-5—Research Version (SCID [22]) and medical assessment at the baseline visit [15, 23]. T1DE-London: the electronic health records were searched using a pre-determined checklist aligning with the STEADY assessment. Psychiatric, psychosocial and psychotherapeutic formulations were extracted from anonymised clinic letters (T1DE-London) and from the SCID interview data and narrative reports (STEADY baseline assessment), expanded by a checklist for T1DE-specific behaviours informed by previous qualitative studies amended to the categories (Table 3) [4, 6, 13, 24]. The extracted data were summarised in initial case vignettes, using a predefined structure. The vignettes were anonymised and shortened before content analysis and discussion with collaborators, and further revised after internal multidisciplinary team discussions to ensure each vignette was appropriately adjusted so that participants could not be identified by any characteristics, while retaining important biomedical and psychosocial information. The 70 anonymised T1DE vignettes are presented in the electronic supplementary material (ESM).

### Phenotype generation from vignettes

The research team developed prototypes of the three main T1DE phenotypes emerging from clusters of T1DE-specific behaviours and psychiatric comorbidity (inductive approach) and integration of existing theoretical frameworks (deductive approach) [6, 13, 24].

The prototypes were presented in a multidisciplinary workshop (on 21 March 2023) of a local network of healthcare professionals with special interest and experience in treating T1DE (psychiatrists, diabetologists, diabetes specialist nurses, dietitians, health psychologists, clinical psychologists, researchers, people with lived experience of type 1 diabetes; *n*=8). The phenotypes and examples of case vignettes for each of the phenotypes, and case vignettes that were not clearly falling into one of the three categories, were discussed. The workshop participants had different professional backgrounds and experiences (liaison psychiatry, physician, CBT, eating disorder psychiatry, health psychology, clinical psychology, mixed-methods research, epidemiology, lived experience of type 1 diabetes). The 1.5 h discussion was held in a hybrid format (in person/Microsoft Teams), and was recorded and transcribed for content analysis. The main outcomes were that the three phenotypes had clinical face-value but needed refining of nomenclature (e.g. should T1DE insulin-omission subtype be called ‘diabulimia’, or ‘insulin omission’?) and further differentiation of type 1 diabetes-specific disordered eating/eating disorder and established eating disorder behaviours.

### Phenotype refinement in iterative multidisciplinary process

The T1DE phenotypes were further refined following the feedback from the workshop, to introduce sub-categories and provide more clarity on the defining behaviours/clinical features for each one. An international multidisciplinary workshop of a network of healthcare professionals with special interest and experience in treating T1DE (psychiatrists, diabetologists, diabetes specialist nurses, dietitians, health psychologists, clinical psychologists from UK, USA, Denmark; *n*=16) was held (21 September 2023) using videoconferencing. The T1DE phenotypes were presented, alongside some vignettes and cases presented by workshop participants to see how they would be defined in the new framework. An initial 1 h presentation and discussion, followed by a 30 min breakout session to discuss cases and a 30 min group discussion summarising the discussions, were recorded, transcribed and analysed for content. An online collaborative platform was used for polling (e.g. ‘which phenotype do you think this case aligns with and why?’) and commenting. The T1DE phenotypes were presented at national conferences (Diabetes UK annual conference, 27 April 2023, and Royal College of Psychiatry Faculty of Eating Disorders Spring Conference, 29 April 2024, UK) to receive feedback from the target audience working in diabetes and in eating disorder specialisms.

The feedback from the international workshop and the conferences was used by the multidisciplinary research team to simplify the phenotypes’ descriptions and to branch out into sub-groups within the three main phenotypes and to describe clinical severity stages, to meet the requests for simplification and applicability to future clinical pathway development.

#### Grouping of vignettes into phenotypes

Vignettes were categorised into phenotypes independently by the study psychiatrist (DP) and diabetologist (MS). The phenotype allocations and clinical severity scoring for each case were rated independently and then discussed case by case to resolve inter-rater differences. This was an iterative process, for example, if inter-rater differences brought to light that an additional phenotype subtype description would be needed to further describe clusters of phenotypes (e.g. hypo subtypes), all 70 vignettes were re-analysed against this new subtyping.

#### Clinical severity scoring

Additional considerations around the conceptualisation of risk emerged from the multidisciplinary discussions. Separating medical and psychiatric risk was felt to be perpetuating an unhelpful narrative about how and who should be supporting people with T1DE. Furthermore, this would overlook the inherent bidirectional relationship between diabetes and mental health in both the development and maintenance of T1DE. Hence, we developed a combined risk-assessment tool as an iteration of a previously developed biomedical T1DE risk score in the T1DE-London project [14], using the vignette series to inform the integrations of medical and psychiatric risk factors and to generate a unified risk rating. Vignettes were categorised into clinical severity scoring independently by the study psychiatrist (DP) and diabetologist (MS). This was an iterative process, for example, when it became apparent that substance misuse was present in a large proportion of cases that also displayed clinical high risk, this factor was added to the risk score and all 70 vignettes were re-analysed in the adapted scoring system.

## Results

### Participant characteristics, mental health comorbidity and T1DE-specific behaviours/cognitions

The 70 participants (94% women) were 34.5 ± 10.6 years old and had a diabetes duration of 18.4 ± 12.5 years. Baseline anthropometric and biomedical characteristics are described in Table 1, and psychiatric diagnoses (from SCID and psychiatric clinical assessments) are listed in Table 2. All but one participant fulfilled criteria for a formal eating disorder diagnosis, half of whom met criteria for OSFED, 39% bulimia nervosa, 9% anorexia nervosa and 3% binge eating disorder. Over 50% had a mood disorder, 44.3% had an anxiety disorder and one in five had a trauma-related disorder (Table 2). Prevalences of T1DE-specific cognitions and behaviours are listed in Table 3: fear of weight gain was the most prevalent cognition (94.3%), followed by fear of hypoglycaemia (20.0%). The majority engaged in insulin restriction behaviour (basal 45.7%, bolus 70.0%), 17.1% reported intentional insulin over-administration to invoke hypoglycaemia, more than half were restricting food/carbohydrates and binge eating not covered by insulin was reported by half of the group (Table 3). The individual case vignettes are presented in the ESM.

**Table 1:** Anthropometric and biomedical data and diabetes acute complications rates

Variable	Number of available datasets	Value
Anthropometric data		
Sex at birth	70	
Female		66 (94.3)
Male		4 (5.7)
Gender	70	
Women		65 (92.9)
Men		5 (7.1)
Age (years)	70	35.5 (30.5–48.8)
Ethnic origin	70	
White		52 (74.2)
Black or mixed black		7 (10)
Asian or mixed Asian		3 (4.3)
Unknown		8 (11.4)
Biomedical data		
Diabetes duration (years)	70	18.4 ± 12.5
HbA_1c_ (mmol/mol)	70	84.8 ± 32.9
HbA_1c_ (%)	70	10.2 ± 3.0
BMI (kg/m^2^)	70	26.2 ± 6.4
Diabetes complications acute		
DKA	70	
Participants experiencing one or more DKA episode(s) in the past 12 months		16 (22.9)
1 DKA episode		8
2 DKA episodes		3
>2 DKA episodes		5
Number of DKA episodes per person in past 12 months	70	0 (0–17)
SH		
Participants experiencing one or more SH episode(s) in the past 12 months	65	8 (11.4)
1 SH episode		3
2 SH episodes		4
>2 SH episodes		1
Number of SH episodes per person in past 12 months	65	0 (0–3)

Data are shown as number (%), median (range) or mean ± SD

SH, severe hypoglycaemia

**Table 2:** Mental health diagnoses per DSM-5

Mental health diagnosis per DSM-5 (*N*=70)	Number (%)
Eating disorder	69 (98.6)
Anorexia nervosa	6
Bulimia nervosa	27
Binge eating disorder	2
OSFED	34
Mood disorder	38 (54.3)
Psychotic disorder	0 (0)
Anxiety disorder	31 (44.3)
Trauma and trauma-related disorder	14 (20)
Other DSM-5 diagnosis	21 (30)
Emotionally unstable personality disorder	6 (8.6)
Attention-deficit/hyperactivity disorder	6 (8.6)
Excoriation disorder	2 (2.9)
Trichotillomania	2 (2.9)
Substance misuse	3 (4.3)
Borderline personality disorder	1 (1.4)
Obsessive–compulsive disorder	1 (1.4)
Number of mental health diagnoses per DSM-5 in addition to eating disorder	
No additional mental health diagnosis	17 (24.3)
1 additional mental health diagnosis	19 (27.1)
2 additional mental health diagnoses	19 (27.1)
3 additional mental health diagnoses	13 (18.6)
4 additional mental health diagnoses	2 (2.9)
Combined mood and anxiety disorders	21 (30)

**Table 3:** T1DE-specific cognitions and behaviours

T1DE-specific cognition or behaviour (*N*=70)	Number (%)
Fear of weight gain	66 (94.3)
Fear of hypoglycaemia—insulin avoidance	14 (20)
Needle phobia	2 (2.9)
Insulin restriction—basal	32 (45.7)
Insulin restriction—bolus	49 (70)
Indirect insulin restriction—carb restriction	36 (51.4)
Insulin over-administration, intentionally invoking hypoglycaemia	12 (17.1)
Self-harm with insulin/intentional insulin overdose	2 (2.9)
Not checking blood glucose	9 (12.9)
Food restriction—general, weight and shape	38 (54.3)
Food restriction—general, perfectionism	19 (27.1)
Food restriction—carbohydrate-specific	36 (51.4)
Binge eating triggered by hypoglycaemia	25 (35.7)
Binge eating not covered by bolus	35 (50)
Binging as self-harm	1 (1.4)
Compensation/purging behaviour	
Excessive exercise	4 (5.7)
Purging/vomiting	5 (7.1)
Purging/diuretics	2 (2.9)
Purging/laxatives	2 (2.9)

### Phenotypes and subtypes

The T1DE phenotype names developed here have the acronym T1DE as part of their definition name, to reflect that type 1 diabetes (T1) is equally important as the disordered eating/eating disorder (DE). The main driver of T1DE-related behaviour (insulin omission, food restriction, binge eating) is reflected in the first part of the phenotype names. This was intentional to allow an easy initial differentiation for clinicians. When analysing the vignettes, data saturation was reached, meaning behaviour patterns and phenotype patterns were observed repeatedly while no new patterns emerged.

#### Insulin-omission (diabulimia) T1DE

**Fig. 1:**
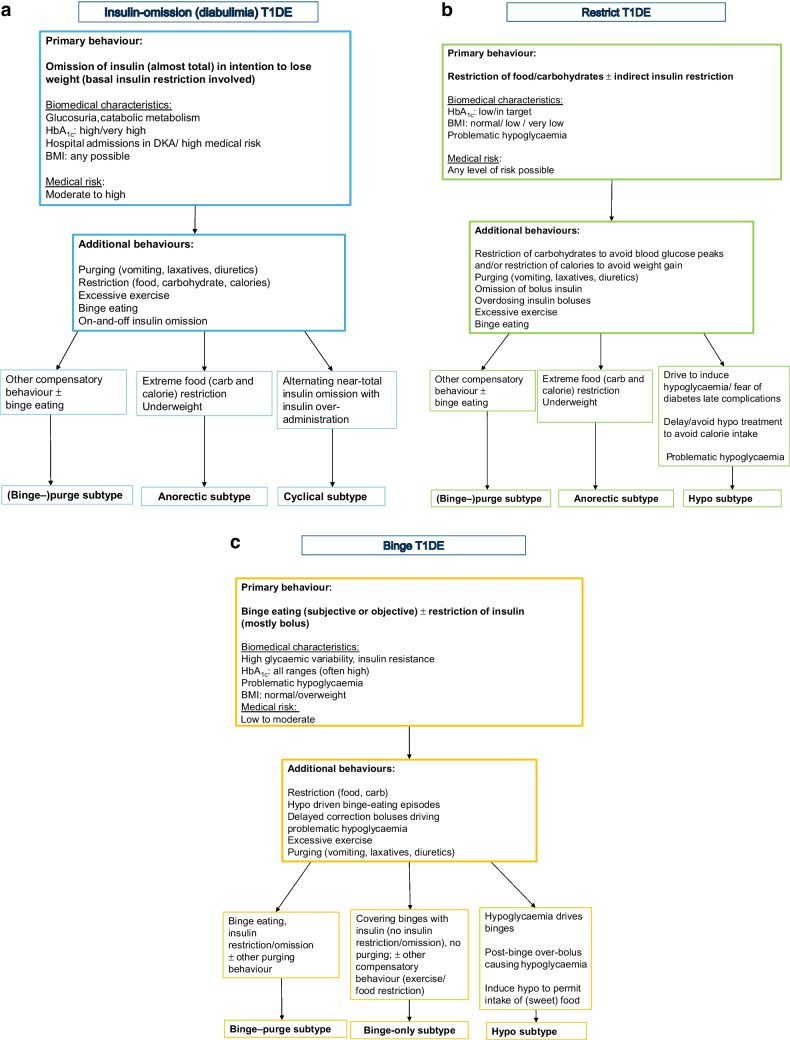
T1DE phenotypes: (**a**) insulin-omission (diabulimia) T1DE, (**b**) restrict T1DE and (**c**) binge T1DE

Insulin-omission (diabulimia) T1DE (Fig. 1a) is primarily identified by the behaviour of insulin omission, with the intention to lose weight or prevent weight gain. Insulin omission focuses on basal insulin (often combined with bolus insulin omission) and can be almost total, with compensatory doses of short-acting insulin given to avoid diabetic ketoacidosis (DKA). There are common biomedical characteristics constituting moderate to high medical risk of a high/very high HbA_1c_, glycosuria, catabolism and hospital admissions with DKA. Body mass index (BMI) ranged from the underweight category (anorectic subtype) to healthy or obese categories (binge–purge and cyclical subtypes). Additional behaviours seen include purging through use of laxatives, vomiting or diuretics; restriction of food, overall food/calorie intake or specifically carbohydrates; excessive/compulsive exercise as a means to control weight and shape; binge eating; and intermittent (cyclical) insulin use. When one of these behaviours was a regular part of the symptom profile, a subtype descriptor was necessary to define the complexity of the presentation. These included: (1) binge–purge subtype if binge eating, with or without other compensatory behaviours, in addition to insulin omission present (insulin restriction remains the dominant behaviour and high HbA_1c_ the predominant medical risk feature, differentiating it from binge T1DE); (2) an anorectic/restrictive subtype if food restriction was evident; and (3) a cyclical subtype where there was a clinical picture of near total insulin omission (over more than 24 h) alternating with insulin over-administration or ‘rescue doses’. Twenty-eight (40%) of the 70 case vignettes fulfilled criteria for insulin-omission T1DE and its subtypes, and, of these, six were identified as insulin-omission (diabulimia) T1DE with no specific subtype, 15 as (binge–)purge subtype, four as anorectic subtype and three as cyclical subtype (Table 4).

**Table 4:** Prevalence of the three T1DE phenotypes in the study participants and prevalence of each risk grade (low, moderate, severe)

T1DE phenotype	Number (%)	Risk grade
Insulin-omission (diabulimia) T1DE	28 (40)	
Insulin-omission, no specific subtype	6	Moderate: 2; severe: 4
Insulin-omission (binge–)purge subtype	15	Moderate: 2; severe: 13
Insulin-omission anorectic subtype	4	Moderate:1; severe: 3
Insulin-omission cyclical subtype	3	Moderate:1; severe: 2
Restrict T1DE	17 (24.3)	
Restrict, no specific subtype	4	Low: 4
Restrict (binge–)purge subtype	6	Low: 1; moderate: 3; severe: 2
Restrict anorectic subtype	2	Severe: 2
Restrict hypo subtype (of which 2 also purge)	5	Low: 2; moderate: 3
Binge T1DE	25 (35.7)	
Binge-only, no specific subtype	2	Moderate: 2
Binge–purge subtype	10	Low: 5; moderate: 5
Hypo subtype	10	Low: 6; moderate: 2; severe: 2
Hypo and binge–purge subtype	3	Low: 1; moderate: 2
Combined medical and risk grade		
Low	19 (27.1)	
Moderate	23 (32.9)	
Severe	28 (40)	

#### Restrict T1DE

Restrict T1DE (Fig. 1b) is characterised primarily by restriction of overall intake of food/calories or carbohydrate-specific foods and the indirect restriction of insulin required as a result of reducing carbohydrates. Biomedical features can include a low or in-target range HbA_1c_, recurrent and/or severe hypoglycaemia (e.g. episodes of severe hypoglycaemia needing third-party assistance) and a healthy or underweight category BMI. Any level of medical risk can be present. Additional behaviours noted were the restriction of carbohydrates to avoid peaks in blood glucose readings on sensor traces; purging through vomiting, laxatives or diuretics; omission of bolus insulin doses (but continuing sufficient amount of basal insulin cover) or taking more insulin than required; excessive or compulsive exercise; and occasional binge eating (but not as a dominant feature). Subtypes were therefore further identified as: (1) binge–purge subtype, where other compensatory behaviour, combined with or without binge eating, is a prominent behaviour and there is no intentional omission of insulin after binging; (2) anorectic subtype, with extreme food restriction and underweight; and (3) the hypo subtype. The hypo subtype was a particular presentation where food/carb restriction or over-correction with insulin following a meal (in fear of high blood glucose levels caused by food driving late complications) results in hypoglycaemia, sometimes evoking problematic hypoglycaemia (e.g. hypoglycaemia unawareness, severe hypoglycaemia), and sometimes presenting with avoidance of hypoglycaemia treatment. Seventeen participants (24.3%) fulfilled criteria for restrict T1DE and its subtypes: four had restrict T1DE with no specific subtype, six had (binge–)purge subtype, two restrict/anorectic subtype and five hypo subtype, two of whom also had purging behaviours and one binging behaviour (Table 4).

#### Binge T1DE

Binge T1DE (Fig. 1c) is characterised by the primary behaviour of subjective or objective binge eating with or without the omission of insulin (mostly bolus insulin, maintaining sufficient basal insulin supply). These presentations often showed high glycaemic variability and, thus, while HbA_1c_ was often high, it could be in any range. BMI was in either the healthy or overweight categories and medical risk was usually low to moderate. Additional behaviours included partial omission of insulin; other compensation through vomiting, laxatives, diuretics or exercise; hypo-driven binges or over-treating binges precipitating hypoglycaemia. Subtypes were identified as: (1) binge–purge, where binge eating was the primary behaviour with compensation through vomiting, laxative or diuretic use (binge eating is the predominant behaviour and insulin may be reduced but to a lesser degree than is the case with the insulin-omission T1DE binge–purge subtype); (2) binge-only subtype with binging covered by insulin and without purging behaviours, with or without other compensatory behaviours such as restriction of food or carbohydrates or excessive exercise; (3) hypo subtype with hypoglycaemia (sometimes caused by intentional insulin administration to ‘allow’ sweet food) as the trigger for binge eating episodes, or postprandial insulin over-treatment of binge eating evoking hypoglycaemia. Twenty-five (35.7%) of the clinical presentations fulfilled criteria for binge T1DE, of which two were in the binge-only subtype, ten in the binge–purge subtype and ten in the hypo subtype, and three showed characteristics of both hypo- and binge–purge subtypes (Table 4).

### Clinical T1DE severity scoring

**Fig. 2:**
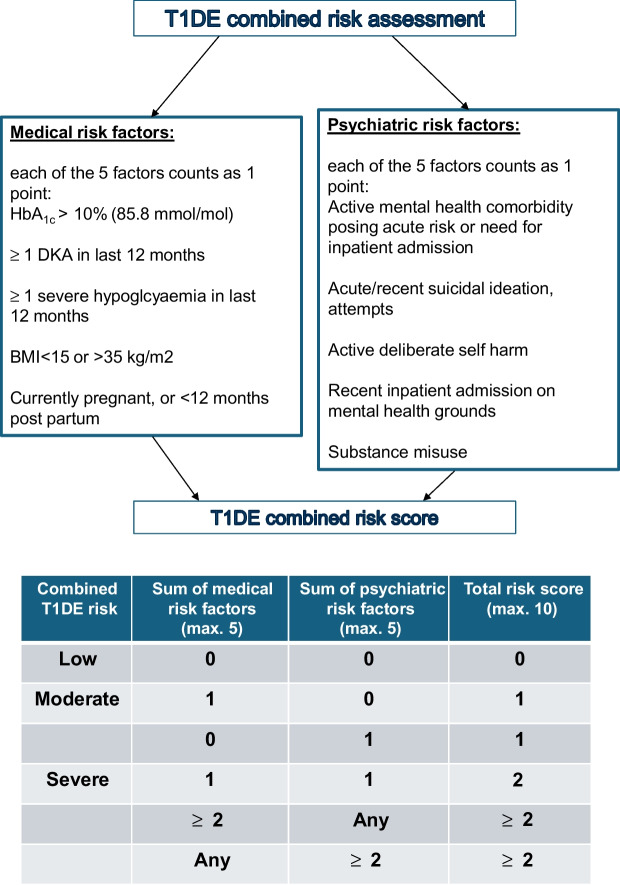
Flow chart for T1DE severity assessment combining medical and psychiatric risk factors

Building upon previous work of this group, we included the five risk parameters of the T1DE severity score used for London-T1DE [14] and assigned one point for each: HbA_1c_ greater than 86 mmol/mol (10%); one or more DKA in the last 12 months; one or more severe hypoglycaemia in the last 12 months; BMI of less than 15 kg/m^2^ or greater than 35 kg/m^2^ [14]; and current pregnancy or up to 12 months in the postpartum period. The five psychiatric risk factors were assigned one point each and included active mental health comorbidities posing acute risk or need for inpatient admission, acute/recent suicidal ideation or attempts, active deliberate self-harm, recent mental health inpatient admission and substance misuse. A total score of zero reflected a low risk; a total score of 1 (either 1 medical or 1 psychiatric risk factor) reflected a moderate combined risk; and a total score of 2 or more was considered high combined risk (Fig. 2). The severity grading showed that 27% were low risk, 33% moderate and 40% severe clinical risk across the entire group, with clusters of higher severity in the insulin-omission T1DE phenotype and a cluster of low severity in the binge T1DE phenotype (Table 4).

## Discussion

This case vignette series of 70 adults living with T1DE captures a broad spectrum of clinical severity and diverse presentations, described in detail from physical and mental health perspectives through a structured multidisciplinary assessment.

Three novel T1DE phenotypes, insulin-omission (diabulimia) T1DE, restrict T1DE and binge T1DE, were generated through an iterative, multidisciplinary process grounded in real-world clinical cases. The most frequently identified phenotype was insulin-omission (diabulimia) T1DE, followed by binge T1DE and restrict T1DE, and clinical severity spanned the full spectrum, with overall higher severity ratings in the insulin-omission (diabulimia) T1DE phenotype and lower severity ratings in the binge T1DE phenotype.

This study represents the first empirical delineation of clinical phenotypes of T1DE, moving beyond the symptom-based categorisations embedded in current eating disorder diagnostic systems. The approach integrates biomedical, psychological and behavioural parameters into a unified clinical taxonomy. This represents a conceptual advance in the field of diabetes psychiatry by operationalising precision mental health within a diabetes framework. Unlike previous questionnaire-driven cluster analyses of disordered eating in diabetes (e.g. [25]), our methodology grounds phenotype definition in real-world clinical data and multidisciplinary synthesis, offering an original translational bridge from qualitative case data to diagnostic frameworks.

This large case series of adults living with T1DE and the granularity of clinical combined medical and psychiatric assessment, as well as the wide and representative spread of clinical presentations and clinical severity, was a robust basis for developing our T1DE phenotypes. Compared with a previous piece of work that was based on Diabetes Eating Problem Survey – Revised (DEPS-R) [26] questionnaire-driven phenotype development [27], this case series provides more depth of clinical understanding of the presentations and the risk associated. With this inductive process of generating the phenotypes from a case series rather than a deduction from a pre-existing psychometric instrument for eating disorders, we were able to capture phenotypes not yet represented in screening questionnaires such as DEPS-R and rarer presentations, such as the cyclical omission of insulin subtype and hypoglycaemia-driven subtypes. Clinical severity stratification was previously described solely based on biomedical parameters (but not psychiatric risk factors) in our London-T1DE project describing the cohort of individuals living with severe T1DE [14], whereas in the current study, we have also integrated the criteria for mild and moderate clinical presentations and the psychiatric risk scoring.

The methodological rigour of this work is grounded in its use of two ethically approved, clinically well-characterised datasets (STEADY and London-T1DE) and its iterative, multidisciplinary analytic process. Triangulation across professional and disciplinary perspectives, and independent sorting of vignettes before consensus discussions, ensured analytic robustness and transparency.

The T1DE phenotype names developed here have the main driver of T1DE-related behaviour (insulin omission, food restriction, binge eating) in their name. Adding the suffix ‘T1DE’ to each of the phenotype names was done to reflect T1DE as a separate presentation compared with a stand-alone eating disorder, by using the acronym T1 for type 1 diabetes and DE for disordered eating/eating disorder, with combined diabetes-specific description and maintenance factors that are not usually included in existing eating disorder diagnoses outside the context of type 1 diabetes. Considering binge eating (perceived loss of control over an eating episode and eating unusually large amounts of food) regardless of objective amount of food consumed as clinically significant within our proposed phenotypes is another notable consideration, binging in the context of type 1 diabetes often being associated with other disordered eating behaviours and emotional distress [28] and the impact on blood glucose levels [5]. The restrict T1DE phenotype is a phenotype not yet described by others [27].

Our proposal of a combined risk tool aids clinicians to conceptualise and communicate risk concerns in assessment and planning of management and additional supports. Risk parameters largely align with those outlined in the MEED guidance (a national guidance under the umbrella of the Royal College of Psychiatry, on the management of eating disorder emergencies) [29]. While the MEED guidance offers extensive detail on medical stabilisation of an eating disorder as well as general psychiatric considerations in extremely unwell individuals [29], our combined tool may offer an accessible, concise option for low to moderate severity presentations in outpatient and primary care settings.

Limitations of this study are the lack of external validation of our findings, under-representation of men and non-white ethnicity groups and the need to meaningfully include people with lived experience of T1DE in refining the phenotype nomenclature. We could not quantify T1DE behaviours related to diabetes management, for example, the degree of insulin omission (type, extent, cyclical patterns); degree of carbohydrate/food restriction; or frequency of binging or purging (in relation to hypoglycaemia or without hypoglycaemia). Furthermore, it is not easy to differentiate what constitutes disordered eating behaviour and what is more likely to be a form of risk taking or lack of diabetes self-care. Differentiated approaches to different T1DE phenotypes will be useful when offering HCL insulin therapy (currently broadly rolled out in the UK), for example, a person with insulin-omission T1DE may need a more gradual transition to HCL, in order to avoid too rapid weight gain and pseudo-hypoglycaemia symptoms, whereas a person with restrict T1DE may need support to avoid further enhancing perfectionist traits that drive carbohydrate and food restriction on the HCL therapy.

Next steps will be to include: (1) lived experience perspectives to refine the wording of the phenotype descriptions and to ensure the categorisations feel accurate to people with lived experience of diabetes and to people working in clinical settings that provide care for people with T1DE; (2) gender- and culture-related aspects; (3) validation of the T1DE phenotypes by developing screening and diagnostic tools for coherent identification in clinical settings and integrating lived experience voices in shaping these tools; and (4) testing the validity of the severity grading. Development of treatment pathways to match the T1DE phenotypes and the severity grading, as well as preventative interventions targeting high-risk groups, such as younger people [30, 31], will be needed.

Manuals for clinicians to apply the diagnostic tools to identify phenotypes of T1DE will need to be developed collaboratively with healthcare professionals in diabetes and in eating disorders, before the T1DE phenotypes can screened for use in clinical practice. The T1DE phenotypes can inform workforce training, improve early identification and facilitate tailored treatment pathways, aligning with current NHS England priorities for integrated diabetes and mental healthcare and with the personalised medicine framework outlined by Pouwer et al [32].

By providing the first structured classification of T1DE phenotypes and an integrated risk-grading tool, this work lays the foundation for developing national screening and diagnostic tools that can guide clinical triage, clinical service commissioning and intervention development.

## Supplementary Information

Below is the link to the electronic supplementary material.

Supplementary file1 (PDF 268 KB)

## Data Availability

The case vignette series the phenotype analysis is based on is shared in the ESM. Source data for the case vignette development cannot be shared for confidentiality reasons. Biomedical and anthropometric data can be shared upon request (corresponding author) for the purposes of systematic reviews and meta-analyses.

## Abbreviations

BMI: Body mass index
CBT: Cognitive behavioural therapy
DEPS-R: Diabetes Eating Problem Survey – Revised
DKA: Diabetic ketoacidosis
DSM-5: Diagnostic and Statistical Manual of Mental Disorders, 5th Edition
HCL: Hybrid closed loop
OSFED: Other specified feeding or eating disorder
SCID: Structured Clinical Interview for DSM-5—Research Version
STEADY: Safe management of with people Type 1 diabetes and EAting Disorders studY
T1DE: Type 1 diabetes and disordered eating
