# Supplementary material for: Clinical and psychological phenotypes of type 1 diabetes and disordered eating derived from a case vignette series: T1DE phenotypes
Source: Diabetologia. 2026 May 26;69(8):2155–67. doi: 10.1007/s00125-026-06756-9 (PMC13310201; doi:10.1007/s00125-026-06756-9)
Supplement: Supplementary file 1 — Supplementary file1 (PDF 268 KB) [file 125_2026_6756_MOESM1_ESM.pdf]

T1DE vignettes

Anonymised vignettes derived from clinical records and study records of the London-T1DE and STEADY projects.

T1DE, type 1 diabetes disordered eating; T1DM, type 1 diabetes; STEADY, Safe management of Type 1 diabetes and Eating Disorder studY; HbA1c, glycated haemoglobin A1c; BMI, body mass index; EUPD, emotionally unstable personality disorder; DKA, admission in diabetic ketoacidosis; ITU, intensive care unit; SSRI, selective serotonine reuptake inhibitor; PTSD, post-traumatic stress disorder; OSFED, other specified feeding or eating disorder; OCD, obsessive compulsive disorder; ADHD, attention deficit hyperactivity disorder

Insulin-omission (diabulimia) T1DE

1) woman, 28 years old

T1DM for 14 years

Disordered eating behaviours began in late teenage years after weight changes around time of T1DM diagnosis. Currently restricts food intake, **insulin omission**, high intensity exercise, misuse of levothyroxine and diuretics. History of binging triggered by hypoglycaemia.

Meets diagnosis for OSFED, engaging in cognitive behavioural therapy, mood closely linked to weight.

HbA_{1c} 13% (118.6mmol/mol) BMI 28.5 kg/m^2

Medical Risk**: multiple admissions for DKA**, HbA_{1c} >10%, bilateral retinopathy, autonomic dysfunction, gastroparesis, neuropathy and insulin oedema. Coeliac disease causing additional distress around food choices.

Psychiatric Risk: recent bullying leading to loss of work.

Diagnostic phenotype: **Insulin-omission (diabulimia) T1DE**

Combined risk- severe

2) woman, 23 years old

T1DM for 8 years

Disordered eating behaviours began around diagnosis which was experienced as chaotic and traumatic. Strong negative association with insulin and weight gain. Has had difficulties managing her diabetes since diagnosis. **Predominant behaviour insulin omission**.

Diagnosed with severe PTSD following multiple traumas and adverse childhood events.

HbA_{1c} 11.9% (106.6mmol/mol) and BMI 23.6 kg/m^2

Medical risk: HbA_{1c} >10%, proliferative retinopathy and DKA requiring recent ITU admission.

Psychiatric risk: severe PTSD (engaged in treatment) and recurrent depression; previous deliberate self-harm and suicidal ideation.

Diagnostic phenotype: Insulin-omission (diabulimia) T1DE

Combined risk- severe

3) woman, 26 years old

T1DM for 15 years

7-year history of **insulin omission for weight control** and laxative misuse.

Meets diagnosis for moderate depression, PTSD, atypical anorexia nervosa and OCD symptoms since her teens- no current treatment. Explains use of hyperglycaemia to numb emotions.

HbA_{1c} 15.7% (148.1mmol/mol) and BMI 22.6 kg/m^2

Medical Risk: **several DKAs in last 12 months**, HbA_{1c} >10%.

Psychiatric risk: untreated mental health comorbidities, ongoing terminal illness of mother affecting mood and anxiety.

Diagnostic phenotype: **Insulin-omission (diabulimia) T1DE**

Combined risk- severe

4) man, 27 years old

T1DM for 4 years

No eating disorder symptoms prior to T1DM diagnosis- developed body image concerns and had difficulties accepting T1DM diagnosis, as insulin therapy caused weight gain. Current symptoms **include insulin omission for weight control**, experiences improved mood and confidence when blood glucose levels are high.

HbA_{1c} 12.3 % (111mmol/mol) and BMI 28 kg/m^2

Medical risk: **1 DKA in 12 months**, HbA_{1c} >10%.

Psychiatric: no acute psychiatric risks.

Diagnostic phenotype: **Insulin-omission (diabulimia) T1DE**

Combined risk- moderate

5) woman, 31 years old

T1DM for 12 years

Disordered eating for 15 years predating T1DM diagnosis and included restriction for weight loss. After T1DM diagnosis, began **omitting insulin** secondary to shame about injecting while on holiday. Now **partially omits insulin (bolus) for weight control, restricts basal insulin, sometimes completely omits basal insulin**, general food restriction, continues to have poor body image and fear of weight gain. Binging triggered by hypoglycaemia but otherwise not binging.

Meets diagnosis for OSFED.

HbA_{1c} 13.5% (115.6mmol/mol) and BMI 22.2 kg/m^2

Medical risk: HbA_{1c} >10%, diabetic retinopathy and autonomic neuropathy (postural hypotension, resting tachycardia, gastroparesis). Also experiences chronic fatigue, migraines, polycystic ovarian syndrome, chronic pain and fibromyalgia.

Psychiatric risk: subthreshold social anxiety disorder, mental health strongly affected by poor diabetes care, no acute psychiatric risks.

Diagnostic phenotype: **Insulin-omission (diabulimia) T1DE**

Combined risk- moderate

50) woman, 31 years old

Initially diagnosed with type 2 diabetes 10 years ago, then with T1DM 7 years ago

Always conscious of weight and shape and strong focus of weight loss and blame after type 2 diabetes diagnosis. Eating disorder behaviours began after T1DM diagnosis and continue to include overeating and intermittent **avoidance of insulin (basal and bolus) to manage weight**.

Meets diagnosis for OSFED

HbA_{1c} 13.4 % (114.5mmol/mol) and BMI 31.5 kg/m^2 (within first year post-partum)

Medical risk: HbA_{1c} >10%, high BMI, 2 DKA (nil since 2020) and no severe hypos.

Psychiatric risk: no acute psychiatric risk.

Diagnostic phenotype: **Insulin-omission (diabulimia) T1DE**

Combined risk- severe (accounting for postpartum status)

6) woman, 23 years old

T1DM for 6 years

5-year history of body image disturbance++, **binging and purging through insulin omission and insulin restriction to control weight**.

Meets diagnosis for depression, previous trauma (sexual assault and bullying). EUPD traits evident (including emotional dysregulation).

HbA_{1c} 13.2% (120.8mmol/mol), BMI 27.5kg/m^2

Medical risk**: 3 DKA in last 12 months** (recent severe DKA requiring ITU), HbA_{1c} >10%.

Psychiatric risk: homelessness, self-neglect, deliberate self-harm and previous suicide attempts with insulin overdose.

Diagnostic phenotype: **Insulin-omission (diabulimia) T1DE, binge-purge subtype**

Combined risk- severe

7) woman, 42 years old

T1DM for 29 years

Over 20 years history of **binging** and recurrent depression predating diabetes. **Insulin omission** began at diagnosis of T1DM then binging and **purging through vomiting and laxatives**.

Meets diagnosis for **bulimia nervosa**- purging, recurrent depression and suicidal ideation, previously treated with cognitive behaviour therapy and SSRI. Insulin pump therapy unsafe use not responding to alarms.

HbA_{1c} 12.0% (107.7mmol/mol) BMI 34 kg/m^2

Medical risk: HbA_{1c} >10%, not checking blood glucose, gives random bolus doses of insulin. No admissions in 12 months but describes **symptoms of hyperglycaemia and ketosis weekly**.

Psychiatric risk: history of suicidal ideation but strong protective factors now in place. History of domestic abuse but currently in supportive marriage.

Diagnostic phenotype: **Insulin-omission (diabulimia) T1DE, binge-purge subtype**

Combined risk- severe

8) woman, 40 years old

T1DM for 27 years

Long-standing depression predating T1DM. Ongoing depression affecting symptoms**- binging** and **purging through insulin omission**, low self-esteem and body image disturbance.

Meets diagnosis for depression and **bulimia nervosa- purging subtype**.

HbA_{1c} 17% (162.3mmol/mol) BMI 20.7 kg/m^2

Medical risk: HbA_{1c} >10%, ischaemic heart disease, chronic kidney disease, diabetic neuropathy, Charcot's Foot.

Psychiatric risk: untreated depression.

Diagnostic phenotype**: Insulin-omission (diabulimia) T1DE, binge-purge subtype**

Combined risk- severe

9) woman, 35 years old

T1DM for 20 years

12-15 years history of **binging and purging through insulin omission** and fear of weight gain.

Meets diagnosis for depression and diabetes distress/burnout.

HbA_{1c} 11.2%(98.9mmol/mol) BMI 24.5 kg/m^2

Medical risk: reports **self-treating multiple perceived DKA at home** (healthcare worker), HbA_{1c} >10% - no hospital admissions, not checking blood glucose with symptomatic hyperglycaemia; diabetic retinopathy (laser treated).

Psychiatric risk: suicidal ideation in last 12 months.

Diagnostic phenotype: **Insulin-omission (diabulimia) T1DE, binge-purge subtype**

Combined risk- severe

10) woman, 40 years old

T1DM for 21 years

Eating disorder behaviours include restriction**, binging, purging through vomiting and insulin omission**; previous laxative use and occasional herbal diuretics. All behaviours have improved and lowest HbA_{1c}; weighs herself multiple times daily.

Drive for perfectionist blood glucose control, intense fear of weight gain interferes with adherence to insulin pump therapy- now on multiple daily injections and uses partner's support to administer insulin.

Meets diagnosis for complex PTSD and anxiety; on melatonin-receptor agonist.

HbA_{1c} 11.2% (98.9mmol/mol) and BMI 24.7 kg/m^2

Medical risk: impaired hypoglycaemia awareness, dental damage from purging and **5 DKAs in last 12 months**, HbA_{1c} >10%, much better motivated since with reduction in behaviours and lowest HbA_{1c} in years.

Psychiatric risk: no acute psychiatric risks.

Diagnostic phenotype: **Insulin-omission (diabulimia) T1DE, binge-purge subtype**

Combined risk- severe

11) woman 27 years old

T1DM for 18 years

Current symptoms include **binge eating, insulin omission**, fear of hypoglycaemia, diabetes distress.

Meets diagnosis for depression, **bulimia nervosa-purging subtype**, **EUPD** diagnosis- under care of community mental health team.

HbA_{1c} 11.8% (105.5mmol/mol) and BMI 27.2 kg/m^2

Medical risk: diabetes complications including maculopathy, retinopathy, nephropathy, neuropathic pain and **one severe hypoglycaemia episode in 12 months**, HbA_{1c} >10%,

Psychiatric risk: recent decline in mental health secondary to family and work stressors.

Diagnostic phenotype: **Insulin-omission (diabulimia) T1DE, binge-purge subtype**

Combined risk- severe

12) woman, 26 years old

T1DM for 14 years

Current symptoms include **binging, insulin omission** (for self-harm and emotional numbing), fear of weight gain and body image disturbance.

Meets diagnosis for **bulimia nervosa, purging**, severe depression, generalised anxiety and panic attacks following diabetes diagnosis and PTSD- multiple traumata.

HbA_{1c} 12.7% (115.3mmol/mol) and BMI 23 kg/m^2

Medical risk: **multiple DKAs**, HbA_{1c} >10%.

Psychiatric risk: **deliberate self-harm, past insulin overdose x 2**, emotional dysregulation, untreated depression.

Diagnostic phenotype: **Insulin-omission (diabulimia) T1DE, binge-purge subtype**

Combined risk- severe

13) woman, 31 years old

T1DM for 15 years

Body image disturbance and low self-esteem predate T1DM diagnosis. Current symptoms include food restriction, **binging, insulin omission fuelled by fear of weight** gain experienced with insulin initiation. Purging through vomiting in the past.

Meets diagnosis for bulimia nervosa-purging and anxiety disorder, significant childhood adversity.

HbA_{1c} 14.0% (129.5mmol/mol) and BMI 23.3 kg/m^2

Medical risk: **one DKA in last 12 months**, HbA_{1c} >10%.

Psychiatric risk: general psychiatric risk with multiple psychiatric diagnoses.

Diagnostic phenotype: **Insulin-omission (diabulimia) T1DE , binge-purge subtype**

Combined risk- severe

14) woman, 24 years old

T1DM for 14 years

10 years history of food restriction, **secret binging and insulin omission for weight loss**.

Anxiety and depression since early teens.

HbA_{1c} 11.7% (104.4mmol/mol) and BMI 24.2 kg/m^2

Medical risk: **10 DKAs in last 12 months**, recently requiring ITU; **multiple severe hypoglycaemic episodes in past** (once with grand mal seizure), HbA_{1c} >10%.

Psychiatric risk: insulin overdoses, history of anxiety, depression, multiple traumas.

Diagnostic phenotype: **Insulin-omission (diabulimia) T1DE, binge-purge subtype**

Combined risk- severe

15) woman, 32 years old

T1DM for 29 years

10 years history of disordered eating- initially over administration of insulin to allow snacking, now **binging and purging through insulin omission, vomiting and laxative** use.

Recurrent depression and anxiety since teenage years- previous medication and therapy

HbA_{1c} 15.7% (148.1mmol/mol) and BMI 29.5 kg/m^2

Medical risk: HbA_{1c}>10%, no DKA, no severe hypoglycaemia, history of recurrent DKA as teenager.

Psychiatric risk: poor self-care, recent strong suicidal ideation with plans.

Diagnostic phenotype: **Insulin-omission (diabulimia) T1DE, binge-purge subtype**

Combined risk- severe

16) woman, 31 years old

T1DM for 7 years

Disordered eating started 7 years ago (after diagnosis)- binging in evenings and weekends. Currently binging and then purging through **bolus insulin omission and basal insulin restriction**, binge eating is also triggered by hypoglycaemia.

Meets diagnosis for OSFED, generalised anxiety disorder and panic disorder, trichotillomania, ADHD, past anorexia nervosa- purging subtype through insulin omission.

HbA_{1c} 13.4% (114.5mmol/mol) and BMI 23kg/m^2

Medical risk: long period of instability with x10 DKA (nil in last 12 mo) and peripheral neuropathy (managed with pregabalin), HbA_{1c} >10%.

Psychiatric risk: untreated ADHD, cannabis use daily, anxiety and depression, multiple adverse childhood events, past suicide attempts (insulin overdose and diazepam overdose).

Diagnostic phenotype: **Insulin-omission (diabulimia) T1DE, binge-purge subtype**

Combined risk- severe

17) woman, 32 years old

T1DM for 25 years

Bulimia nervosa since age 13 (after T1DM diagnosis). **Basal insulin omission as well as binging and purging through insulin omission (bolus) and vomiting and compensating through restriction and exercise**. Currently no vomiting or exercise. Binging also triggered by hypos.

Meets diagnosis for bulimia nervosa, persistent depression since age 9 years, agoraphobia, panic disorder and social anxiety disorder.

HbA_{1c} 9.8 % (77.4mmol/mol) and BMI 31.7 kg/m^2

Medical risk: Multiple DKA in adolescence due to total insulin omission – last admission was over 10 years ago. **Multiple severe hypoglycaemic episodes with one in last 12 months**. Has support from local T1DE service and risk has reduced. Painful peripheral neuropathy.

Psychiatric risk: occasional suicidal thoughts but no plan to act as children are a protective factor.

Diagnostic phenotype: **Insulin-omission (diabulimia) T1DE, binge-purge subtype**

Combined risk- moderate

18) woman, 26 years old

T1DM for 14 years

Current eating disorders behaviours include **binging and purging through vomiting and insulin omission**.

Meets diagnosis for current and persistent major depression, OSFED, Asperger’s syndrome, agoraphobia, separation anxiety, generalized anxiety, trichotillomania.

HbA_{1c} 14% (120.7mmol/mol) and BMI 39 kg/m^2

Medical risk: **HbA_{1c} >10%**, treated retinopathy and peripheral neuropathy. **10 DKAs with 1 in last 12 months** and 1 past severe hypoglycaemia (nil in last 12 months), vitrectomy right eye when pregnant.

Psychiatric risk: **significant mental health comorbidities and multiple emergency department admissions**. She is under community mental health team, requires high support from family and uses crisis services. History of previous overdoses, deliberate self-harm and impulsive behaviour.

Diagnostic phenotype: **Insulin-omission (diabulimia) T1DE, binge-purge subtype**

Combined risk- severe

19) woman, 22 years old

T1DM for 16 years

Currently **binging and purging** through **bolus insulin omission/ basal insulin restriction and carbohydrate- and overall food restriction**, fear of hypoglycaemia. Depression since childhood which occurred in response to adverse life event around time of T1DM diagnosis.

Meets diagnosis for bulimia nervosa, persistent depressive disorder, social anxiety, panic disorder, agoraphobia, past anorexia nervosa requiring inpatient admission.

HbA_{1c} 13.2% (112.5mmol/mol) and BMI 20.4 kg/m^2

Medical risk: **HbA_{1c} >10%**, 8 DKAs and 4 severe hypoglycaemic episodes in the past, but nil in last 12 months, cataract removal, amenorrhea for 1 year with stable weight.

Psychiatric risk: depression since childhood and significant anxiety, **inpatient admission for anorexia nervosa in last 18 months**, significant impact on mental health and functioning since pandemic. History of deliberate self-harm and suicidal ideation.

Diagnostic phenotype: **Insulin-omission (diabulimia) T1DE, binge-purge subtype**

Combined risk-severe

20) woman, 27 years old

T1DM for 6 years

Disordered eating predates diabetes from teenage years. History of anorexia nervosa as a teenager- accessed Children and Adolescents Mental Health Services and completed a course of therapy. Currently **omits short acting insulin, reduces dosis of basal insulin, purging through vomiting after meals** driven by fear of weight gain.

Currently meets diagnosis for OSFED.

HbA_{1c} 11.7% (104.4mmol/mol) and BMI 33.5 kg/m^2

Medical risk: peripheral neuropathy, **HbA_{1c} >10%.**

Psychiatric risk: past intentional overdose as a teenager, no current deliberate self-harm, or suicidal ideation.

Diagnostic phenotype: **Insulin-omission (diabulimia) T1DE, purge subtype**

Combined risk- moderate

21) woman, 36 years old

T1DM for 26 years

Basal and bolus insulin omission, **food restriction**, binging, **purging through vomiting**, intense fear of weight gain related to insulin, entrenched **fear of hypoglycaemia and intentional over administration of insulin**.

Meets diagnosis for **anorexia nervosa- binge/purge subtype**, severe anxiety, recent bereavement, history of sexual assault, past illicit drug use.

HbA_{1c} 10% (85.8mmol/mol) and **BMI 16.2 kg/m^2**

Medical risk: **2 DKAs in last 12 months**, low BMI, **HbA_{1c} >10%**, severe diabetic retinopathy, diabetic nephropathy, peripheral neuropathy affecting day to day function.

Psychiatric risk: **current day patient treatment for eating disorder** and past inpatient admission, social isolation, current suicidal thoughts but strong protective factors.

Diagnostic phenotype: **Insulin-omission (diabulimia) T1DE, anorectic subtype**

Combined risk- severe

22) woman 33 years old

T1DM for 11 years

Disordered eating began after diagnosis due to fear of developing diabetic complications and high morning blood glucose levels**. Restricts insulin directly and indirectly through restricting carbohydrates** particularly in the evening- this helps to regulate emotions, numb anxiety and reduce insulin requirements.

Meets diagnosis of **anorexia nervosa** (engaged with local ED service), depression and anxiety.

HbA_{1c} 14% (129.5 mmol/mol) and **BMI 15 kg/m^2**

Medical risk: low BMI, **HbA_{1c} >10%,** recurrent DKA and **recent DKA requiring ITU** admission, recent cataract surgery.

Psychiatric risk: one previous inpatient admission for eating disorder and refusing further admission.

Diagnostic phenotype: **Insulin-omission (diabulimia) T1DE, anorectic subtype**

Combined risk- severe

23) woman, 29 years old

T1DM for 14 years

10 years history of eating disorder with symptoms including fear of weight gain, **restriction of calories and carbs**, **fear of insulin as weight gaining driving insulin omission** and excessive exercise.

Meets diagnosis for **atypical anorexia nervosa.**

HbA_{1c} 15.6% (147mmol/mol) and **BMI 19.1 kg/m^2**

Medical risk: diabetic retinopathy and neuropathy, **HbA_{1c} >10%**, no DKA, no severe hypoglycaemia.

Psychiatric risk: previous psychiatric inpatient admission.

Diagnostic phenotype: **Insulin-omission (diabulimia) T1DE, anorectic subtype**

Combined risk- severe

24) woman, 27 years old

T1DM for 11 years

Disordered eating began after T1DM diagnosis and includes current symptoms of **restriction of general food intake, compulsive exercise and omission of insulin (basal and bolus)**.

Meets diagnosis for OSFED- **atypical anorexia nervosa** and ADHD. Past diagnosis of panic disorder.

HbA_{1c} 17% (162.3mmol/mol) and BMI 22.9 kg/m^2

Medical risk: **HbA_{1c} >10%,** peripheral neuropathy secondary to diabetes, no severe hypoglycaemia or DKA.

Psychiatric risk: no acute psychiatric risk.

Diagnostic Phenotype: **Insulin-omission (diabulimia) T1DE, anorectic subtype**

Combined risk- moderate

25) woman, 22 years old

T1DM for 10 years

Current symptoms include **insulin restriction, on-and off pattern of omission of long-acting insulin, over administration of insulin as deliberate self-harm** (hyperglycaemia allows brain fog and emotional avoidance) and occasional binge eating.

Meets diagnosis for OSFED, severe depression, anxiety and unstable **EUPD**. Poor diabetes care since diagnosis, as family not supportive.

HbA_{1c} 14.1% (130.6mmol/mol) and BMI 20.2 kg/m^2

Medical Risk: **recent DKA and 2 severe hypoglycaemia episodes**, **HbA_{1c} >10%**.

Psychiatric Risk: severe unstable mental health, **deliberate self-harm through insulin over administration** (previously punching), past inpatient admission under the Mental Health Act.

Diagnostic phenotype: **Insulin-omission (diabulimia) T1DE, cyclical subtype**

Combined risk- severe

26) woman, 28 years old

T1DM for 11 years

Post-partum depression following birth of twins triggered **binging, insulin omission, vomiting and laxative** for weight control **in a cyclical pattern**.

Meets diagnosis for severe depression.

HbA_{1c} 10.8% (94.5mmol/mol) and BMI 31.1 kg/m^2

Medical risk**: 2 DKAs in 12 months and several (3) severe hypoglycaemic episodes**, background retinopathy, maculopathy and peripheral neuropathy, **HbA_{1c} >10%**.

Psychiatric risk: significant emotional abuse from parental figures and severe depression predate post-partum depression.

Diagnostic Phenotype: **Insulin-omission (diabulimia) T1DE, cyclical subtype**

Combined risk- severe

27) woman, 31 years old

T1DM for 25 years

Started binge/purging in secondary school (age 14) and omitting insulin. Current **binging and omitting insulin in a cyclical manner with occasional vomiting**.

Current meets diagnosis for **bulimia nervosa**, current agoraphobia and panic disorder, meets diagnosis for body dysmorphic disorder, excoriation disorder, significant history of abuse, past major depression.

HbA_{1c} 12.3% (103.2 mmol/mol) and BMI 23.1 kg/m^2

Medical risk: **HbA_{1c} >10%**, history of 5 DKA and 4 severe hypoglycaemic episodes (in context of alcohol), none in last 12 months, 4 seizures in last 1.5 years now stable on medication, treated retinopathy, sight impaired, necrobiosis lipoidica diabeticorum since childhood.

Psychiatric risk: multiple mental health comorbidities and under Community Mental Health team.

Diagnostic phenotype: **Insulin-omission (diabulimia) T1DE, cyclical subtype**

Combined risk- moderate

Restrict-T1DE

30) woman, 42 years old

T1DM for 33 years

Disordered eating, social anxiety and low mood in teenage years. In her early 20's started to **control eating by restriction** and then will tip into **subjective binging a few times a month**. Currently restricts daily intake of calories.

Meets diagnosis for **OSFED** and anxiety as well as past social anxiety disorder.

HbA_{1c} 6.5% (43.5mmol/mol) and BMI 23.9 kg/m^2

Medical risk: Multiple DKA's and severe hypoglycaemic episodes prior to last 12 months.

Psychiatric risk: subthreshold anxiety and low mood, no acute psychiatric risks.

Diagnostic phenotype: **Restrict T1DE**

Combined risk- low

31) woman, 26 years old

T1DM for 4 years

Disordered eating started after diagnosis. Lost weight in lead up to diagnosis and following diagnosis, focused on gaining weight and treating diabetes. Was diagnosed with polycystic ovarian syndrome and diet advice given around food clashed with diabetes structured education recommendations. Past depressive episode treated with sertraline. At the start of the pandemic, exercise increased and started **experiencing guilt if didn't exercise and when treating hypos**. Currently subjective binging, exercise to compensate, **restricting carbs, chewing and spitting and under dosing insulin**.

Meets diagnosis for **OSFED**.

HbA_{1c} 6.5% (43.5mmol/mol) and BMI 20.5 kg/m^2

Medical risk: no DKA, no severe hypoglycaemia.

Psychiatric risk: no acute psychiatric risks.

Diagnostic phenotype: **Restrict T1DE**

Combined risk- low

33) woman, 30 years old

T1DM for 15 years

Disordered eating began around T1DM diagnosis. **Currently restricts carbohydrates and fats to reduce insulin and avoid weight gain**, **omits bolus insulin, excessive exercise** and subjective overeating.

Meets diagnosis for **OSFED**.

HbA_{1c} 8.9% (68mmol/mol) and BMI 24.3 kg/m^2

Medical risk: background retinopathy, no DKA, x1 severe hypo in 2022.

Psychiatric risk- nil acute.

Diagnostic phenotype: **Restrict T1DE**

Combined risk- low

34) woman, 25 years old

T1DM for 23 years

Past history of anorexia nervosa (restricting subtype) with **general restriction of calories, over-exercise** and **indirect insulin restriction through carbohydrate restriction**. Changed to insulin pump therapy age 9 years due to eating disorder. Fluctuating mood and anxiety in context of anorexia nervosa but not reaching diagnostic level. Currently restricting carbohydrates and fats- **perfectionistic control of blood sugar**.

Meets diagnosis for **OSFED**.

HbA_{1c} 6.5% (43.5mmol/mol) and BMI 18.6 kg/m^2 (and stable)

Medical risk: Hashimoto's thyroiditis, convulsions secondary to severe hypoglycaemia as a child, no DKA, no diabetic complications.

Psychiatric risk- previous inpatient treatment for anorexia nervosa.

Diagnostic phenotype: **Restrict T1DE**

Combined risk- low

35) woman, 21 years old

T1DM for 6 years

History of calorie restriction and depression prior to T1DM diagnosis; **food restriction, insulin omission**, **laxative use** and **excessive exercise**.

Meets diagnosis for depression and OSFED; history of being bullied, needle phobia.

HbA_{1c} 9.9% (84.7mmol/mol) and BMI 21 kg/m^2

Medical risk: **2 severe hypoglycaemia episodes in 12 months**.

Psychiatric risk: **recent severe depressive episode with suicidal ideation**, history of being bullied and difficult family dynamics, transitioning from child to adult eating disorder services.

Diagnostic phenotype: **Restrict T1DE, purge subtype**

Combined risk- severe

36) woman, 31 years old

T1DM for 13 years

Depression and disordered eating predate T1DM diagnosis. Symptoms include **food restriction, insulin omission and vomiting**.

Meets diagnosis for **OSFED**, severe depression, **EUPD** and needle phobia.

HbA_{1c} 7.3% (56.3mmol/mol) and BMI 25.9 kg/m^2

Medical risk: **1 DKA in 12 months**, Chron's disease.

Psychiatric risk: past admission following insulin overdose, childhood trauma (sexual and physical).

Diagnostic phenotype: **Restrict T1DE, purge subtype**

Combined risk- moderate

37) woman, 28 years old

T1DM for 14 years

Disordered eating onset 18 months ago and progressed to **severe anorexia requiring inpatient treatment**; **restriction of calories, carbohydrate specific restriction, insulin omission (bolus), exercise, purging and use of diet pills**.

Meets diagnosis for **OSFED** and history of social anxiety disorder, panic disorder and trichotillomania.

HbA_{1c} 6.9% (51.9mmol/mol) and BMI 23.4 kg/m^2

Medical risk: unable to respond to hypoglycaemia during assessment (refused to take treatment).

Psychiatric risk: acute **suicidal ideation**, risk of rapid weight loss and unable to engage in crisis management planning.

Diagnostic phenotype: **Restrict T1DE, purge subtype**

Combined risk-high

38) woman, 41 years old

T1DM for 3 years

Disordered eating began in childhood- dieting and exercise for weight control. Current **restriction of carbohydrates and overall intake to reduce insulin, skipping meals to avoid insulin** and nocturnal hypos, **binging every 2-3 weeks** with compensation through restriction, underdosing insulin (bolus) and **exercise and using laxatives**.

Meets diagnosis for **OSFED**, ADHD.

HbA_{1c} 9.3% (72.3mmol/mol) and BMI 24 kg/m^2

Medical risk: no DKA, no severe hypoglycaemia.

Psychiatric risk: no acute psychiatric risks.

Diagnostic phenotype: **Restrict T1DE, binge-purge subtype**

Combined risk-low

39) woman, 25 years old

T1DM for 8 years

Partially treated depression and anxiety pre-dating T1DM diagnosis. Disordered eating began after T1 diagnosis. Mood and ED behaviours continue to be affected by T1DM. Past restriction and purging (meeting anorexia nervosa criteria). Currently **general food and carbohydrate restriction**, **binging, purging through insulin omission and occasional vomiting and laxative use** and binging also triggered by hypoglycaemia.

Meets diagnosis for **bulimia nervosa**- purging, past anorexia nervosa, persistent depressive disorder, generalized anxiety disorder and panic attacks, social anxiety disorder, body dysmorphic disorder, excoriation disorder.

HbA_{1c} 9.5% (74.4mmol/mol) and **BMI 18.2 kg/m^2**

Medical risk: 5 DKA in past, none in last 12 months, osteopenia, background retinopathy, altered bowel habit, and no severe hypoglycaemia.

Psychiatric risk: Impulsive overdose age 13/14 and past deliberate self-harm (cutting)- no passive suicidal ideation.

Diagnostic phenotype: **Restrict T1DE, binge-purge subtype**

Combined risk- moderate

40) woman, 41 years old

T1DM for 10 years

ED behaviours predate diagnosis of T1- restricting in teens, chewing and spitting in adolescence, counting calories, excessive exercise. Low mood and anxiety also predate diabetes. Current behaviours include **daily restriction of carbohydrates**, **subjective binge eating** not covered by bolus, **insulin restriction (basal and bolus)**.

Currently meets criteria for **OSFED**, current depression, social anxiety with history of anorexia nervosa, bulimia nervosa, recurrent depression, specific phobia, panic disorder.

HbA_{1c} 8.9% (68.2mmol/mol) and BMI 21.2 kg/m^2

Medical risk: long covid, **1 DKA in last 12 months**, no severe hypoglycaemia, multiple admissions for DKA in the past.

Psychiatric risk: multiple admissions for eating disorders in past- during one admission admits to suicidal ideation but no attempts and no deliberate self-harm. **Significant psychiatric comorbidity past and present** would raise risk threshold in light of coping mechanisms and emotional regulation.

Diagnostic phenotype: **Restrict T1DE, binge-purge subtype**

Combined risk: moderate

29) woman, 28 years old

T1DM for 27 years (since 18mo old) – parental management of diabetes until 2 years ago

2-year history of weight loss in context of depression (neglected diabetes care) triggered by a traumatic miscarriage; progressed to **insulin omission, calorie restriction and episodes of overeating not covered by insulin**. Currently intermittently omitting insulin and not checking blood glucose.

Meets diagnosis for depression, anxiety, **EUPD**, history of sexual assault and past deliberate self-harm.

HbA_{1c} 9.4% (79.2mmol/mol) and **BMI 14.7 kg/m^2**

Medical risk: **low BMI**, **pregnant** (multiple previous miscarriages), **2 DKA admissions in last 2 years and a recent severe hypoglycaemic episode**. Multiple past DKAs, severe hypoglycaemic episodes (seizures) and an ITU admission with cerebral oedema.

Psychiatric risk: poor engagement with perinatal mental health services.

Diagnostic phenotype: **Restrict T1DE, anorectic subtype**

Combined risk- severe

41) man, 23 years old

T1DM for 15 years

6-year history of symptoms beginning around time of DAFNE (diabetes structured education programme in the UK) and insulin pump therapy start and increased stress at school- **food and fluid restriction, purging through vomiting and excessive exercise**.

Diagnosis of **anorexia nervosa** requiring recent inpatient admission.

HbA_{1c} 8.4% (68.3mmol/mol) **BMI 15.2 kg/m^2** (13.9 kg/m^2 on referral)

Medical risk: restricts food and fluids, **2 severe hypos in 12 months**, low BMI.

Psychiatric risk: **recent psychiatric inpatient admission**.

Diagnostic phenotype: **Restrict T1DE, anorectic subtype**

Combined risk -severe

42) woman, 28 years old

T1DM for 6 years

Disordered eating started at 17 years and included **food restriction and exercise for weight loss**, which progressed to **cycles of restriction during the week and binging on weekends**. After T1DM diagnosis, started restricting overall calories and carbohydrates (indirect insulin restriction), compulsive exercise, no binging but eats large amounts of artificially sweetened foods, **intentionally invokes hypoglycaemia and then over treats**. Strong perfectionistic tendencies.

Meets diagnosis for **OSFED**, generalised anxiety disorder, emetophobia & excoriation disorder.

HbA_{1c} 5.2% (30.1mmol/mol) and BMI 25.5 kg/m^2

Medical risk: PCOS, no DKA, no severe hypoglycaemia.

Psychiatric risk: no acute psychiatric risks.

Diagnostic phenotype: **Restrict T1DE, hypo subtype**

Combined risk-low

43) woman, 40 years old

T1DM for 28 years

Disordered eating began shortly after diagnosis (coincided with transition to secondary school) and included binging and restricting cycles then binging and insulin omission in university with significant weight loss. Current symptoms include fear of weight gain, **calorie and carbohydrate restriction** with **subjective binging cycles, bolus insulin omission**, **fear of hypos, intentionally invoking hypos, hypos trigger subjective binging**, seeking **adjunct diabetes medication for additional weight loss** and perfectionistic tendencies.

Meets diagnosis for major depressive disorder, OSFED, OCD, trichotillomania as well as past generalised anxiety disorder, illicit drug use and subclinical persistent depressive disorder.

HbA_{1c} 11.8% (98mmol/mol) and BMI 24.3 kg/m^2

Medical risk: **HbA_{1c} >10%**, no DKA, no severe hypoglycaemia.

Psychiatric risk: multiple mental health comorbidities, no acute psychiatric risks.

Diagnostic phenotype: **Restrict T1DE, purge and hypo subtypes**

Combined risk- moderate risk

44) woman, 51 years old

T1DM for 46 years

Bulimia nervosa- purging subtype since teenage years, then binge drinking in her 20’s and occasional binging and purging of food, laxative use, exercise and restriction for weight control in her 30’s. Currently **restricts calories and carbohydrates, perfectionistic blood sugar control, occasional non-carbohydrate binges**. **Invokes hypoglycaemia to justify eating**, **hypoglycaemia trigger binging** but will adjust hypoglycaemia treatment to avoid blood sugar spike.

Meets diagnosis for **OSFED**.

HbA_{1c} 6.8% (46.5mmol/mol) and BMI 29,7 kg/m^2

Medical risk: resting tachycardia, no DKAs, 5 severe hypoglycaemic episodes in past, long covid syndrome.

Psychiatric risk- no acute psychiatric risk, but past deliberate self-harm by punching self and history of depression.

Diagnostic phenotype: **Restrict T1DE, binge-purge and hypo subtypes**

Combined risk- low

28) woman, 50 years old

T1DM for 8 years

Past use of diet pills, laxatives and excessive exercise. Current symptoms include **carbohydrate restriction to indirectly restrict insulin**.

Meets diagnosis for depression, panic attacks and alcohol misuse. **Past history of anorexia nervosa** diagnosed in teenage years.

HbA_{1c} 8.3% (62 mmol/mol) and BMI 19.8 kg/m^2

Medical risk: infrequent checking of blood glucose, overcorrects with insulin, **frequent hypoglycaemic episodes, some severe**.

Psychiatric risk: alcohol misuse, one previous episode of deliberate self-harm.

Diagnostic phenotype: **Restrict T1DE, hypo subtype**

Combined risk- moderate

32) woman, 56 years old

T1DM for 51 years

Disordered eating behaviours started after weight loss in context of an infection and included restricting carbs to reduce insulin required. **Currently restricting food intake for weight and shape** as well as **restricting carbohydrates to reduce insulin**, some compulsion to exercise, **fear of hypoglycaemia** and will **skip meals after treating hypos**.

Meets diagnosis for **OSFED**, generalized anxiety disorder and ADHD.

HbA_{1c} 8.9% (68.2mmol/mol) and BMI 24.9 kg/m^2

Medical risk: **reduced hypoglycaemia awareness, severe hypoglycaemia just over a year ago**.

Psychiatric risk: ADHD untreated, generalised anxiety disorder, frequent passive suicidal ideation, 1 insulin overdose aged 25 and 1 additional attempt in past.

Diagnostic phenotype: **Restrict T1DE, hypo subtype**

Combined risk- moderate

Binge- T1DE

45) woman, 30 years old

T1DM for 6 years

Binge eating disorder predates T1DM diagnosis**. Currently binges 4-7 x weekly**, runs sugars high due to **fear of hypos** and denies insulin omission for weight loss.

Meets diagnosis for **binge eating disorder**, major depression, social anxiety and panic disorder.

HbA_{1c} 8.6% (65.1mmol/mol) and **BMI 45.6kg/m^2**

Medical risk: high BMI, no severe hypos or DKA, necrobiosis lipoidica diabeticorum.

Psychiatric risk: no acute psychiatric risk.

Diagnostic phenotype: **Binge T1DE, binge-only subtype**

Combined risk-moderate

46) woman, 46 years old

T1DM since age 1 years

Disordered eating and binge eating on/off since the age of 8 triggered by low mood and life stressors. No compensatory behaviours.

Meets diagnosis for **binge eating disorder** and major depressive disorder (recurrent since adolescence).

HbA_{1c} 7.4% (52.7mmol/mol) and **BMI 40.4 kg/m^2**

Medical risk: high BMI, no recent severe hypoglycaemic episodes or DKAs (x 1 DKA and severe hypoglycaemia in past), no diabetes complications.

Psychiatric risk: poor self-care.

Diagnostic phenotype: **Binge T1DE, binge-only subtype**

Combined risk- moderate

47) trans man, 21 years old

T1DM for 5 years

History of deliberate self-harm through scratching and head banging since age 14 years. **Binging 1-2 times per month, bolus insulin omission, fear of hypoglycaemia, underdosing insulin**. Previous restriction for weight and shape and restriction of carbohydrates.

Meets criteria for **OSFED**, past anxiety disorder and depression.

HbA_{1c} 7.3% (51.7mmol/mol) and BMI 20.1 kg/m^2

Medical risk: No diabetes complications, no severe hypoglycaemic episodes or DKA.

Psychiatric risk: past history of head banging and deliberate self-harm via high blood glucose levels, one intentional overdose with insulin 2 years ago.

Diagnostic phenotype: **Binge T1DE, binge-purge subtype**

Combined risk- low

48) woman, 32 years old

T1DM for 3 years

Disordered eating began after diagnosis. Initially experienced weight gain with commencing insulin therapy and difficulty with food choices. Initially food avoidance which led to binging in secret. Currently **binging daily and compensates by restriction, laxative misuse, carb specific restriction and exercise**. Denies insulin omission.

Meets diagnosis for **Bulimia Nervosa**, Social Anxiety & Panic Disorder (in relation to perceived diabetes stigma).

HbA_{1c} 11.1% (90.8mmol/mol) BMI 29.9kg/m^2

Medical risk: **HbA_{1c} >10%**, no diabetic late complications, no severe hypoglycaemic episodes or DKAs

Psychiatric risk: no acute psychiatric risk.

Diagnostic phenotype: **Binge T1DE, binge-purge subtype**

Combined risk- moderate

49) woman, 52 years old

T1DM for 15 years

Eating disorder behaviours began after T1DM diagnosis- **binging and insulin omission (bolus)**, also short acting insulin omission outside of binging for weight control and fear of weight gain.

Meets diagnosis for **bulimia nervosa**, previously treated with cognitive behaviour therapy and SSRI

HbA_{1c} 12.5% (113.1mmol/mol) and BMI 32.5 kg/m^2

Medical Risk: **HbA_{1c} >10%**, no diabetic late complications, no severe hypoglycaemic episodes or DKAs

Psychiatric risk: no acute psychiatric risks.

Diagnostic phenotype: **Binge T1DE, binge-purge subtype**

Combined risk- moderate

51) woman, 25 years old

T1DM for 4 years

Disordered eating predates T1DM diagnosis. Started restricting food and exercising for weight loss 7 years ago. Currently **restricts carbohydrates and calories for weight loss and in order to reduce insulin requirement and then tips into binging**. Under doses insulin after binging and tends to over-exercise. History of chronic dieting, laxative misuse and fat binder use.

Meets diagnosis for **OSFED**.

HbA_{1c} 7.9% (57.9mmol/mol) and BMI 28.7 kg/m^2

Medical risk: no diabetic late complications, no severe hypoglycaemic episodes or DKAs, recurrent urinary tract infections.

Psychiatric risk: signed off work for burnout, anxiety and stress which worsens binging. No acute psychiatric risks.

Diagnostic phenotype: **Binge T1DE, binge-purge subtype**

Combined risk- low

52) woman 48 years old

T1DM for 29 years

Disordered eating began with binging at age of 13/14 years (views binging on junk food as form of deliberate self-harm), depression and generalised anxiety disorder age 16 years. Currently **cycles through binging and restriction**, **underdoses insulin when binges**, **restricts food to keep blood glucose in range** and reduce insulin requirements, binging triggered by hypoglycaemia.

Meets criteria for **OSFED**, current persistent depression, generalised anxiety disorder and ADHD.

HbA_{1c} 7.5 (53.8mmol/mol) and BMI 22.3 $kg/^2$

Medical risk: no recent severe hypoglycaemic episodes or DKAs (4 severe hypos 5 years ago), diabetic neuropathy (gastroparesis, peripheral neuropathy), cerebral palsy.

Psychiatric risk: past passive suicidal ideation, headbanging when mood low when younger. history of trauma- currently undergoing Eye Movement Desensitisation and Reprocessing therapy.

Diagnostic phenotype: **Binge T1DE, binge-purge subtype**

Combined risk-low

53) woman, 60 years old

T1DM 31 years old

Disordered eating/ comfort eating started after leaving home, then became subjective binging. Initially diagnosed with gestational diabetes and then with T1DM, **started binge eating after having children and omitted insulin to compensate**. Currently binging with insulin avoidance or underdosing and **skipping of meals**, binging also triggered by hypoglycaemia.

HbA_{1c} 7.2% (50.7mmol/mol) and BMI 26.5 kg/m^2

Meets diagnostic criteria for **OSFED**, past depressive disorder and past generalised anxiety disorder.

Medical risk: no severe hypoglycaemic episodes or DKAs, hyperlipidaemia.

Psychiatric risk: **deliberate self-harm by stabbing self with pen** on thighs when distressed and under influence of alcohol.

Diagnostic phenotype: **Binge-T1DE, binge-purge subtype**

Combined risk- moderate

54) woman, 33 years old

T1DM for 18 years

History of anorexia nervosa prior to T1DM diagnosis. Currently **binging, compensates with insulin omission, also calorie and carbohydrate restriction**.

Meets diagnosis for current **bulimia nervosa**, past anorexia nervosa, current major depressive disorder.

HbA_{1c} 10% (79.5mmol/mol) and BMI 23.7kg/m^2

Medical risk: **HbA_{1c} >10%**, history of seizures with severe hypoglycaemia (as a teenager), recurrent DKA (20 episodes) as a teenager, hypothyroidism.

Psychiatric risk: history of deliberate self-harm/intentional overdose in response to insulin omission.

Diagnostic phenotype: **Binge T1DE, binge-purge subtype**

Combined risk- moderate

55) woman, 38 years old

T1DM for 6 years

Disordered eating, currently **binging every 2-3 weeks, compensates through insulin omission (bolus), exercise, restriction of overall calories and carbohydrates for weight and shape and restriction of insulin**.

Meets diagnosis for OSFED, trichotillomania, past depression.

HbA_{1c} 9.8% (77.4mmol.mol) and BMI 31.6 kg/m^2

Medical risk: no severe hypoglycaemic episodes or DKA.

Psychiatric risk: previous 1 day admission and working with crisis team when mood was low and had suicidal ideation. No acute current psychiatric risk.

Diagnostic phenotype: **Binge T1DE, binge-purge subtype**

Combined risk- low

56) woman, 43 years old

T1DM for 7 years

Long history of all or nothing dieting with episodic binging and vomiting. Then started omitting insulin for weight control. Currently emotional **overeating, binging, purges through insulin omission/reduction**, fear of weight gain. **Binge eating in response to hypos**.

Meets diagnosis for bulimia nervosa- purging.

HbA_{1c} 11.3% (92.9mmol/mol) and BMI 25.2 kg/m^2

Medical risk: high HbA1c, hypothyroidism, no severe hypoglycaemic episodes or DKA.

Psychiatric risk: No acute psychiatric risk.

Diagnostic phenotype: **Binge T1DE, binge-purge subtype**

Combined risk- moderate

57) woman, 37 years old

T1DM for 17 years

Disordered eating started after T1DM diagnosis- received treatment for **bulimia nervosa-purging**. Currently **binging, vomiting, restricting carbohydrates to reduce insulin** and binging triggered by hypos.

Meets diagnosis bulimia nervosa-purging, diabetes burnout in past

HbA_{1c} 6.9% (47.6mmol/mol) and BMI 32 kg/m^2

Medical risk: No diabetes complications, no severe hypoglycaemic episodes or DKA.

Psychiatric risk: past suicidal ideation following break up, no acute psychiatric risk.

Diagnostic phenotype: **Binge T1DE, binge-purge subtype**

Combined risk- low

58) woman, 32 years old

T1DM for 24 years

Fear of weight gain, **binging triggered by hypoglycaemia**. Complex interplay between diabetes burnout, depression as primary event affecting diabetes and depression as secondary to poor diabetes care.

Meets criteria for **OSFED with past binge eating disorder**, past recurrent depression, past persistent depression. Likely undiagnosed ADHD.

HbA_{1c} 7.8% (56.8mmol/mol) and BMI 23.9 kg/m^2

Medical risk: 10 DKAs in past, no recent severe hypoglycaemic episodes or DKAs

Psychiatric risk: past suicidal ideation, no acute psychiatric risk

Diagnostic phenotype: **Binge T1DE, hypo subtype**

Combined risk- low

59) woman, 38 years old

T1DM for 36 years

Disordered eating since childhood, initially as result of anxiety (avoiding eating in front of others, vomiting), discovered insulin avoidance would subjectively reduce anxiety. Later started cycles of restriction and compulsive exercise which would tip into binging. Current behaviours include **binge eating with restriction** (overall and carbohydrate specific) or **overdosing of insulin to induce hypoglycaemia which triggers binge eating**.

Meets diagnosis for **bulimia nervosa-purging**, persistent depression with **severe major depressive episodes**, generalised anxiety disorder, past panic disorder, multiple overdoses in the past (insulin and paracetamol) with involuntary inpatient admissions.

HbA_{1c} 6.6% (44.5mmol/mol) and BMI 27.5kg/m^2

Medical risk: **2 severe hypoglycaemic episodes in 12 months** and not treating adequately.

Psychiatric risk: significant mental health comorbidities, **multiple overdoses in past** (insulin and paracetamol).

Diagnostic phenotype: **Binge T1DE, hypo subtype**

Combined risk-severe

60) man, 33 years old

T1DM for 12 years, diagnosis received during ongoing traumatic life event

Body image disturbance, **binging in response to hypos, fear of hypoglycaemia**, **binging and purging through vomiting and insulin omission**.

Meets diagnosis for **bulimia nervosa- purging**, emotional dysregulation, untreated depression, trauma and anxiety, harmful use of alcohol increasing hypoglycaemia risk.

HbA_{1c} 10.7% (93.5mmol/mol) and BMI 36 kg/m^2

Medical risk: **HbA_{1c}>10%, BMI>35 kg/m2** (referred for bariatric surgery), no DKA, no severe hypoglycaemia.

Psychiatric risk: untreated mental health difficulties, harmful use of alcohol, no acute psychiatric risks.

Diagnostic phenotype: **Binge T1DE, hypo subtype**

Combined risk- severe

61) man, 56 years old

T1DM for 28 years

Disordered eating with long history of weight difficulties since childhood (age 7). Met criteria for bulimia nervosa at age 30 years (after T1DM diagnosis). Currently **low frequency binging compensating with restriction and exercise**; carbohydrate restriction with indirect insulin restriction, **intentional invoking of hypos and binges triggered by hypos**.

Meets diagnoses for OSFED, past bulimia nervosa and major depression

HbA_{1c} 6.2% (40.4mmol/mol) and BMI 29.7 kg/m^2

Medical risk: no severe hypoglycaemic episodes or DKAs, no diabetes complications.

Psychiatric risk: no acute psychiatric risks.

Diagnostic phenotype: **Binge T1DE, hypo subtype**

Combined risk- low

62) woman, 44 years old

T1DM for 36 years

Eating disorder behaviours predate diabetes and started around age 15 years. Currently meets diagnosis for **bulimia nervosa- binging and compensating through general and carbohydrate restriction**, exercise and diet pills with history of vomiting and laxatives**. Allows self to go into hypoglycaemia to allow sweet treats**.

Meets diagnosis for bulimia nervosa, past major depressive disorder, specific phobia

HbA_{1c} 6.4% (42.4mmol/mol) BMI 26 kg/m^2

Medical risk: no diabetes complications, no severe hypoglycaemic episodes or DKA in last 12 months (multiple severe hypoglycaemic episodes in past).

Psychiatric risk: no acute psychiatric risk.

Diagnostic phenotype: **Binge T1DE, hypo subtype**

Combined risk- low

63) woman, 56 years old

T1DM for 14 years

Eating disorder began as a teenager- **binge eating disorder** and now **binging in response to hypoglycaemia**, underdoses insulin and restricts carbohydrates and general intake.

Currently meets diagnosis for **OSFED**, depression (feels strongly linked to burden of diabetes) past difficulties with alcohol. Past diagnosis of EUPD- no longer meets diagnosis.

HbA_{1c} 7.4% (52.7mmol/mol) and BMI 33 kg/m^2

Medical risk: 1 DKA, but none in last 12 months, no severe hypoglycaemia, no diabetes complications.

Psychiatric risk: past deliberate self-harm and 2 intentional overdoses age 32 years; one episode of suicidal ideation in the last year- currently well supported and low risk.

Diagnostic phenotype: **Binge T1DE, hypo subtype**

Combined risk- low

64) woman, 57 years old

T1DM for 36 years

Bulimia nervosa predates T1DM diagnosis. Currently **overeats and skips bolus insulin, restricts carbs on/off to reduce insulin**, **hypoglycaemia triggers binging**.

Meets diagnosis for **OSFED**, PTSD current, past bulimia nervosa.

HbA_{1c} 9.5% (74.4mmol/mol) and **BMI 35 kg/m^2**

Medical risk: high BMI, diabetic complications including autonomic neuropathy (altered bowel habit) and peripheral neuropathy (tingling), 2 severe hypoglycaemic episodes in context of alcohol over 10 years ago, no DKA. History of breast cancer and autoimmune gastritis, pernicious anaemia.

Psychiatric risk: no acute psychiatric risk.

Diagnostic phenotype: **Binge T1DE, hypo subtype**

Combined risk- moderate

65) woman, 57 years old

T1DM for 54 years

Disordered eating began 30 years ago with excessive exercise and food restriction. Current behaviours include **subjective binging, hypoglycaemia trigger binging**, compensatory overall and carbohydrate specific restriction, perfectionistic control of blood sugar. History of self-induced vomiting.

Meets criteria for **OSFED**.

HbA_{1c} 6.4% (42.4mmol/mol) and BMI 27.5 kg/m^2

Medical risk: no DKA or severe hypoglycaemic episodes in last 12 months (3 DKA in past), no diabetes complications.

Psychiatric risk: intentional insulin overdose age 25 years.

Diagnostic phenotype: **Binge T1DE, hypo subtype**

Combined risk- low

66) woman, 34 years old

T1DM for 6 years (initially diagnosed as having type 2 diabetes)

Disordered eating predates diabetes diagnosis with binging and restricting. Current **binging**, overall restriction. **Carbohydrate restriction** driven by weight and shape concerns as well to restrict insulin and for diabetes control. **Insulin over administration to invoke hypoglycaemia** and **fear of hypoglycaemia**.

Meets diagnosis for **bulimia nervosa- non purging**, past depression, current specific phobia, complex PTSD (decreasing symptoms).

HbA_{1c} 7% (48.6 mmol/mol) and **BMI 41.4 kg/m^2**

Medical risk: high BMI, no severe hypoglycaemic episodes or DKA.

Psychiatric risk: history of cutting, scratching as **deliberate self-harm** and 2 intentional overdoses in the past.

Diagnostic phenotype: **Binge T1DE, hypo subtype**

Combined risk- moderate

67) woman, 34 years old

T1DM for 32 years

Currently disordered eating behaviours include **binging daily and compensation through calorie restriction and indirect insulin restriction** through carbohydrate restriction as well as **binging triggered by hypoglycaemia**.

Meets diagnosis for **bulimia nervosa**, major depression on medication, generalised anxiety disorder, ADHD. Long history of loss since childhood impacting mood and sense of self.

HbA_{1c} 7.5% (53.8mmol/mol) and BMI 34.4 kg/m^2

Medical risk: hypothyroidism, 4 DKA and 4 severe hypoglycaemic episodes, but nil in last 12 months.

Psychiatric risk: no acute psychiatric risk, multiple mental health comorbidities controlled with medication.

Diagnostic phenotype: **Binge T1DE, hypo subtype**

Combined risk- low

68) woman, 53 years old

T1DM for 47 years

Disordered eating since childhood- secretive eating which progressed to restriction, excessive exercise, binging with vomiting and insulin omission. Symptoms have fluctuated over the years. Currently **subjective binges and underdosing or delays insulin and food restriction to compensate**, carbohydrate specific food restriction. **Binging triggered by hypoglycaemia**.

Meets diagnostic criteria for **OSFED**, generalised anxiety disorder, past major depression and past persistent depression.

HbA_{1c} 8.5% (64.1mmol/mol) and BMI 31.8 kg/m^2

Medical risk: high BMI, no recent severe hypoglycaemic episodes or DKA, diabetes complications (treated retinopathy, coronary heart disease), severe hypoglycaemic episodes in teenage years; severe asthma and long covid syndrome.

Psychiatric risk: impulsive overdose with insulin age 13 years, but nil since. No current acute psych risk.

Diagnostic phenotype: **Binge T1DE, binge-purge and hypo subtypes**

Combined risk- low

69) woman, 33 years old

T1DM for 7 years

Disordered eating with binge eating started following T1DM diagnosis (initially diagnosed during pregnancy). Currently **binging and purging daily through insulin omission (bolus)**, cycles through carbohydrate restriction and **binging triggered by hypoglycaemia**.

Meets diagnosis for **bulimia nervosa- purging**, mild-mod depression and past PTSD.

HbA_{1c} 11.8% (98mmol/mol) and BMI 28.8 kg/m^2

Medical risk: **HbA_{1c} >10%**, some background retinopathy, peripheral neuropathy, 1 DKA and severe hypoglycaemia while in hospital during inpatient admission for autoimmune condition.

Psychiatric risk: no acute psychiatric risk.

Diagnostic phenotype: **Binge T1DE, binge-purge and hypo subtypes**

Combined risk-moderate

70) woman, 30 years old

T1DM for 23 years

Disordered eating after T1DM diagnosis initially with restriction followed by binging and purging, then diabetes burnout led to insulin omission. Binging and purging through insulin omission and vomiting over the years plus binging on alcohol, recreational and illicit drugs. Currently **binging, purging with insulin omission, carbohydrate restriction with indirect insulin restriction**, **binging triggered by hypoglycaemia** and **fear of hypoglycaemia**.

Meets diagnosis for bulimia nervosa, Emotionally Unstable Personality Disorder. Past history of Major Depression, anxiety, PTSD and past alcohol misuse.

HbA_{1c} 7.9% (57.9mmol/mol) and BMI 21.9kg/m^2

Medical risk: History of multiple DKA's and severe hypoglycaemia (none in the past 12 months), diabetic complications (retinopathy, gastroparesis, peripheral neuropathy).

Psychiatric risk: unstable **EUPD**, **recent suicidal ideation** and was able to access crisis services, past insulin OD and ongoing recreational drug use.

Diagnostic phenotype: **binge T1DE, binge-purge and hypo subtypes**

Combined risk- moderate
